# Impact of Modifiable Bleeding Risk Factors on Major Bleeding in Patients With Atrial Fibrillation Anticoagulated With Rivaroxaban

**DOI:** 10.1161/JAHA.118.009530

**Published:** 2020-02-21

**Authors:** Paulus Kirchhof, Sylvia Haas, Pierre Amarenco, Susanne Hess, Marc Lambelet, Martin van Eickels, Alexander G. G. Turpie, A. John Camm, the XANTUS Investigators*

**Affiliations:** ^1^ Institute of Cardiovascular Sciences UHB and Sandwell & West Birmingham Hospitals NHS Trusts University of Birmingham United Kingdom; ^2^ University Heart and Vascular Center Hamburg Hamburg Germany; ^3^ Formerly Technical University of Munich Munich Germany; ^4^ Department of Neurology and Stroke Centre Paris‐Diderot‐Sorbonne University Paris France; ^5^ Medical Affairs Bayer AG Berlin Germany; ^6^ Chrestos Concept GmbH & Co KG Essen Germany; ^7^ Department of Medicine McMaster University Hamilton Ontario Canada; ^8^ Cardiovascular and Cell Sciences Research Institute and Cardiology Clinical Academic Group St George's, University of London London United Kingdom

**Keywords:** anticoagulation, independent predictor, major bleeding, modeling study, modifiable risk factor, Arrhythmias, Atrial Fibrillation, Anticoagulants

## Abstract

**Background:**

Reducing major bleeding events is a challenge when managing anticoagulation in patients with atrial fibrillation. This study evaluated the impact of modifiable and nonmodifiable bleeding risk factors in patients with atrial fibrillation receiving rivaroxaban and estimated the impact of risk factor modification on major bleeding events.

**Methods and Results:**

Modifiable and nonmodifiable risk factors associated with major bleeding events were identified from the XANTUS (Xarelto for Prevention of Stroke in Patients With Atrial Fibrillation) prospective registry data set (6784 rivaroxaban‐treated patients). Parameters showing univariate association with bleeding were used to construct a multivariable model identifying independent risk factors. Modeling was used to estimate attributed weights to risk factors. Heavy alcohol use (hazard ratio [HR]=2.37; 95% CI 1.24–4.53); uncontrolled hypertension (HR after parameter‐wise shrinkage=1.79; 95% CI 1.05–3.05); and concomitant treatment with antiplatelets, nonsteroidal anti‐inflammatory drugs, or paracetamol (HR=1.80; 95% CI 1.24–2.61) were identified as modifiable, independent bleeding risk factors. Increasing age (HR=1.25 [per 5‐year increment]; 95% CI 1.12–1.38); heart failure (HR=1.97; 95% CI 1.36–2.86); and vascular disease (HR=1.91; 95% CI 1.32–2.77) were identified as nonmodifiable bleeding risk factors. Overall, 128 (1.9%) patients experienced major bleeding events; of these, 11% had no identified bleeding risk factors, 50% had nonmodifiable bleeding risk factors only, and 39% had modifiable bleeding risk factors (with or without nonmodifiable risk factors). The presence of 1 modifiable bleeding risk factor doubled the risk of major bleeding.

**Conclusions:**

Elimination of modifiable bleeding risk factors is a potentially effective strategy to reduce bleeding risk in atrial fibrillation patients receiving rivaroxaban.

**Clinical Trial Registration:**

URL: http://www.clinicaltrials.gov. Unique identifier: NCT01606995.

**Clinical Perspective**

**What Is New?**

- In patients with atrial fibrillation treated with rivaroxaban, heavy alcohol use, uncontrolled hypertension, and concomitant treatment with antiplatelets, nonsteroidal anti‐inflammatory drugs, or paracetamol were identified as modifiable, independent bleeding risk factors; increasing age, heart failure, and vascular disease were identified as nonmodifiable bleeding risk factors.
- Thirty‐nine percent of patients who experienced major bleeding events had at least 1 modifiable bleeding risk factor (most of whom also had additional nonmodifiable risk factors).
- The presence of 1 or more of the 3 independent modifiable bleeding risk factors identified in this analysis was associated with a 2‐fold increase in the risk of major bleeding.

**What Are the Clinical Implications?**

- Eliminating or reducing modifiable bleeding risk factors (eg, heavy alcohol use, uncontrolled hypertension, and concomitant therapy with antiplatelets, nonsteroidal anti‐inflammatory drugs, or paracetamol) in the context of integrated atrial fibrillation care may be an effective strategy to reduce the risk of bleeding in anticoagulated patients with atrial fibrillation.

Oral anticoagulation with vitamin K antagonists or non‐vitamin K antagonist oral anticoagulants (NOACs)^1^ prevents stroke and prolongs life in patients with atrial fibrillation (AF).^1^, ^2^, ^3^, ^4^ Although most patients benefit from oral anticoagulation, all anticoagulants increase the risk of major bleeding, including fatal events. Several bleeding risk factors (eg, higher age or prior stroke)^5^, ^6^, ^7^, ^8^, ^9^ cannot be modified and also identify patients with AF at high risk of stroke. Others, such as concomitant therapy with antiplatelet agents or uncontrolled hypertension, are modifiable and offer opportunities to reduce bleeding risk.^3^ Although recent guidelines on the management of AF recommend treating modifiable bleeding risk factors,^3^ the impact of such factors on outcomes in anticoagulated patients with AF has never been quantified. Furthermore, bleeding risk factors in patients treated with NOACs may be different from bleeding risk factors in patients treated with vitamin K antagonists. In this analysis, modifiable and nonmodifiable risk factors of major bleeding were identified in an unselected cohort of AF patients treated with the NOAC rivaroxaban, and the potential maximum benefits of reducing modifiable risk factors were modeled.

## Methods

The authors declare that all supporting data are available within the article and its online supplementary files.

### Patients and Outcome Definitions

The XANTUS (Xarelto for Prevention of Stroke in Patients With Atrial Fibrillation) data set was analyzed; XANTUS, a safety study mandated by the European Medicines Agency, was a real‐world, prospective, observational registry that enrolled unselected adult patients (aged ≥18 years) with AF who had been newly prescribed rivaroxaban for stroke/systemic embolism prevention in routine clinical practice.^10^ Patients who did not provide informed consent were ineligible and contraindications were considered according to the product label. Dose and duration of rivaroxaban therapy were solely at the discretion of the prescribing physician. The study received all appropriate approval by Health Authorities, independent Ethics Committees, and Independent Review Boards. All participants gave written informed consent to participate in the study.^10^ All bleeding events reported by the investigators were analyzed and adjudicated centrally as major or nonmajor based on predefined criteria in accordance with the International Society on Thrombosis and Haemostasis definition of major bleeding. An event was considered treatment emergent if it started on or after the day of the first dose of rivaroxaban and up to 2 days after the last dose. Only treatment‐emergent major bleeding events were used in the analysis because nonmajor bleeding events may be less accurately recorded in an observational setting, and are less likely to be as clinically relevant; bleeding events occurring after discontinuation of rivaroxaban are unlikely to be treatment related. Patient characteristics, including comorbidities and potential risk factors for bleeding events, were recorded as assessed by the treating physician at the initial screening visit before enrollment in the study, except for creatinine clearance (CrCl) and weight (used for body mass index calculation), which were first available values, recorded at any time during the study. Definitions for hypertension, heart failure, and vascular disease are summarized in Table S1. Concomitant medications taken at the time of commencing rivaroxaban therapy and initiated any time after the start of rivaroxaban therapy were recorded during the initial and follow‐up visits, respectively. After the initial screening visit, patients were followed up at ≈3‐month intervals for up to 1 year or until 30 days after permanent discontinuation (if <1 year) of rivaroxaban treatment.

### Statistical Methods

All analyses were based on the safety analysis set, which included all patients exposed to at least 1 dose of rivaroxaban during the observation period. Potential risk factors were explored using separate univariate Cox proportional hazard models, which included all patients with available data for each risk factor assessed. The univariate analyses included both continuous and categorical versions of a variable where applicable. Risk factors with a univariate *P*<0.10 were considered as candidates. In case of high correlation among factors (assessed using Kendall's tau), candidates were removed based on medical judgment. Additional risk factors with *P*≥0.10 were chosen as candidates for the multivariable model selection procedure for medical reasons. Medical judgment was also used to decide whether continuously measured variables were included as continuous (ie, age) or categorical variables (ie, first available CrCl <50 mL/min versus ≥50 mL/min; first available weight >60 kg versus ≤60 kg) in the multivariable model. The risk factors selected after the univariate analyses (Table S2) were included into a multivariable Cox regression model. A backward elimination with a significance level of *P*=0.10 for keeping variables in the model was performed to identify a model with multiple risk factors. Patients with missing values were not included (ie, a multiple imputation model was not performed). The proportional hazards assumption of the Cox model and linearity of age were assessed graphically (Figures S1 and S2). A sensitivity analysis was carried out in which missing values for CrCl were imputed. The model fit of the final multivariable model was assessed visually using a calibration plot (Figure S3),^11^ and model discrimination was assessed using Harrell's C statistic (Table S3).^11^ An internal validation of the model was performed (ie, the C‐statistic corrected for optimism was calculated via the bootstrap technique [200 samples])^11^; parameter estimates were adjusted with parameter‐wise shrinkage factors, and corresponding hazard ratios (HRs) were determined.^12^

The identified risk factors in the final multivariable model were divided into modifiable and nonmodifiable, and the impact of the modifiable risk factors was assessed by comparing the model‐predicted probabilities of major bleeding over time for selected types of patients by showing modeled Kaplan–Meier curves for patients with or without modifiable risk factors and by computing partial population‐attributable risks. The partial population‐attributable risks give the maximum proportion of events that could theoretically be prevented if a specific risk factor (and any associated pathologies) were completely eliminated/reversed. It is applicable in cases where there is more than 1 risk factor of interest and when the set of risk factors includes both modifiable and nonmodifiable risk factors.^13^

Paulus Kirchhof and the co‐authors had access to all the data in the study. Paulus Kirchhof takes responsibility for its integrity and data analysis. The study sponsor oversaw data management and statistical analyses to adhere to Good Clinical Practice standards, while the lead statistician oversaw programming and validation of the statistical analyses.

## Results

Treatment‐emergent major bleeding events occurred in 128 patients over a mean treatment duration of 329 days (2.1 events per 100 patient‐years). Compared with patients without major bleeding events, those with major bleeding events were older; more of them had heart failure and vascular heart disease; and they were more often treated with concomitant antiplatelets, nonsteroidal anti‐inflammatory drugs (NSAIDs), or paracetamol (Table 1). Overall, 118 patients (1.9 events per 100 patient‐years) died and, of these, 12 (0.2 per 100 patient‐years) died of a bleeding event (Table 2). Baseline characteristics of patients who had major bleeding events or died, compared with patients not experiencing these outcomes, are included in Table S4. The mean treatment duration (follow‐up) (± SD) was 329 (±115) days, with a median treatment duration (interquartile range) of 366 (343–379) days. In total, 4223/6784 (62.2%) patients were treated for 1 year.

**Table 1:** Baseline Demographics and Clinical Characteristics of Patients With and Without Treatment‐Emergent Major Bleeding Events in XANTUS

	All Patients (N=6784)	Patients With Major Bleeding (n=128)	Patients Without Major Bleeding (n=6656)	*P* Value
Age, y, mean±SD	71.5±9.95	75.9±9.35	71.4±9.94	<0.001
<75 y, n (%)	3975 (58.6)	52 (40.6)	3923 (58.9)	<0.001
≥75 y, n (%)	2809 (41.4)	76 (59.4)	2733 (41.1)	<0.001
Male, n (%)	4016 (59.2)	81 (63.3)	3935 (59.1)	0.3457
Body mass index, kg/m^2^, mean±SD	28.3±4.98	28.1±5.25	28.3±4.98	0.5769
First available creatinine clearance, n (%)	···	···	···	0.0246
<80 mL/min	2961 (43.6)	87 (68.0)	2874 (43.2)	···
≥80 mL/min	1491 (22.0)	27 (21.1)	1464 (22.0)	···
Missing	2332 (34.4)	14 (10.9)	2318 (34.8)	···
Hepatic insufficiency, n (%)^a^	137 (2.0)	6 (4.7)	131 (2.0)	0.0303
Rivaroxaban dose (first documented), n (%)	···	···	···	0.0004
15 mg	1410 (20.8)	39 (30.5)	1371 (20.6)	···
20 mg	5336 (78.7)	86 (67.2)	5250 (78.9)	···
Other/missing	38 (0.6)	3 (2.3)	35 (0.5)	···
Concomitant ASA or NSAID, n (%)	1118 (16.5)	33 (25.8)	1085 (16.3)	0.0042
Concomitant dual antiplatelets, n (%)	105 (1.5)	5 (3.9)	100 (1.5)	0.0291
Concomitant antiplatelet, NSAID, or paracetamol, n (%)	1363 (20.1)	41 (32.0)	1322 (19.9)	0.0007
Concomitant CYP3A4 or P‐gp inhibitors, n (%)^b^	1313 (19.4)	40 (31.3)	1273 (19.1)	0.0006
Concomitant paracetamol, n (%)	191 (2.8)	9 (7.0)	182 (2.7)	0.0036
Active cancer, n (%)	105 (1.5)	3 (2.3)	102 (1.5)	0.4614
Prior bleeding, n (%)	49 (0.7)	1 (0.8)	48 (0.7)	0.9366
Ulcerative gastrointestinal disease, n (%)	27 (0.4)	1 (0.8)	26 (0.4)	0.4869
Uncontrolled hypertension, n (%)	275 (4.1)	8 (6.3)	267 (4.0)	0.2034
Prior stroke, n (%)	935 (13.8)	22 (17.2)	913 (13.7)	0.2598
Prior MI, n (%)	688 (10.1)	19 (14.8)	669 (10.1)	0.0752
Heart failure at baseline, n (%)	1265 (18.6)	45 (35.2)	1220 (18.3)	<0.0001
Platelet count <80 000, n (%)	39 (0.6)	2 (1.6)	37 (0.6)	0.3577
Diabetes mellitus, n (%)	1333 (19.6)	31 (24.2)	1302 (19.6)	0.1890
Vascular disease, n (%)	1685 (24.8)	55 (43.0)	1630 (24.5)	<0.0001
Heavy alcohol use, n (%)	54 (0.8)	3 (2.3)	51 (0.8)	0.0791
Anemia/reduced hemoglobin, n (%)	203 (3.0)	6 (4.7)	197 (3.0)	0.2558
Known coagulopathy, n (%)	19 (0.3)	1 (0.8)	18 (0.3)	0.2787
Bridging therapy during interruptions, n (%)	100 (1.5)	11 (8.6)	89 (1.3)	<0.0001

The baseline demographics and clinical characteristics from patients in the XANTUS (Xarelto for Prevention of Stroke in Patients With Atrial Fibrillation) study were stratified according to the presence or absence of major bleeding. ASA indicates acetylsalicylic acid; CYP3A4, cytochrome P450 3A4; MI, myocardial infarction; NSAID, nonsteroidal anti‐inflammatory drug; P‐gp, P‐glycoprotein.

a Defined as “abnormal liver function” by the study investigator.

b Strong, moderate, and weak inhibitors were included.

**Table 2:** Characteristics of Patients Who Died of a Bleeding Event in XANTUS

Patient	Event	Concomitant Cardiovascular Conditions
1: 73 y old	ICH, 7 mo after rivaroxaban start (15 mg od)	Hypertension
2: 85 y old	ICH, 14 d after rivaroxaban start (15 mg od)	Prior TIA, CHF, vascular disease, hypertension
3: 74 y old	ICH, 6 mo after rivaroxaban start (15 mg od)	Vascular disease, diabetes mellitus, hypertension
4: 80 y old	ICH, 4 mo after rivaroxaban start (20 mg od)	CHF
5: 60 y old	ICH, 9 mo after rivaroxaban start (20 mg od)	Obesity, CHF, hypertension
6: 63 y old	ICH, 8 mo after rivaroxaban start (20 mg od)	Hypertension
7: 66 y old	ICH, 10 mo after rivaroxaban start (20 mg od)	CHF, vascular disease, hypertension
8: 70 y old	Extracranial bleeding, 3 mo after rivaroxaban start (20 mg od)	CHF, hypertension
9: 76 y old	GI bleeding, 11 mo after rivaroxaban start (20 mg od)	Anemia and prior hemorrhoidal bleeding, CHF, vascular disease, diabetes mellitus, obesity
10: 56 y old	Rectal bleeding, 4 mo after rivaroxaban start (20 mg od)	Prior systemic embolism, CHF, hypertension, obesity
11: 74 y old	Intra‐alveolar hemorrhage, 6 wks after rivaroxaban start (15 mg od)	Hypertension
12: 87 y old	Aortic aneurysm rupture, 10 mo after rivaroxaban start (15 mg od)	CHF, vascular disease, hypertension

All patients had atrial fibrillation and were treated with rivaroxaban. CHF indicates congestive heart failure; GI, gastrointestinal; ICH, intracranial hemorrhage; od, once daily; TIA, transient ischemic attack; XANTUS, Xarelto for Prevention of Stroke in Patients With Atrial Fibrillation.

**Figure 1:**
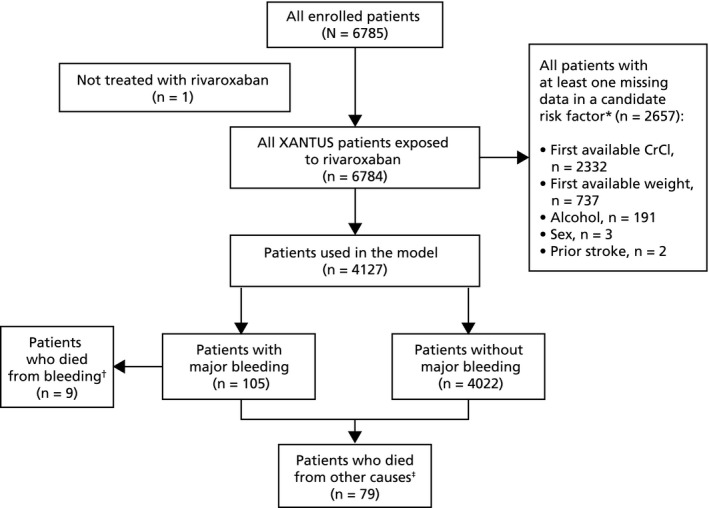
Flow chart of patients in this analysis (STROBE format). *Patients can have missing data in more than 1 candidate risk factor. ^†^Patients who died (treatment‐emergent) with bleeding as cause of death based on model population. ^&ddagger;^Patients who died (treatment‐emergent) excluding bleeding as cause of death based on model population. CrCl indicates creatinine clearance.

**Figure 2:**
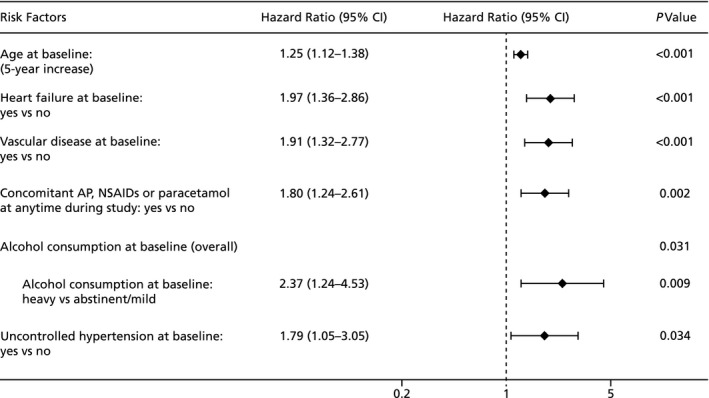
Risk factors associated with major bleeding events in XANTUS (Xarelto for Prevention of Stroke in Patients With Atrial Fibrillation). Forest plot showing all factors associated with major bleeding events in the XANTUS population after parameter‐wise shrinkage. Alcohol consumption was defined as: abstinent (0 g alcohol/d); mild (<40 g alcohol/d); moderate (40–80 g alcohol/d); or heavy (>80 g alcohol/d). AP indicates antiplatelet; NSAID, nonsteroidal anti‐inflammatory drug.

The multivariable analysis included 4127 patients with all data for candidate risk factors available (Figure 1). Compared with patients excluded from the model, patients who were included had a similar mean age and a similar proportion of them were elderly (aged ≥75 years old); however, they were more likely to have heart failure or vascular disease and, as expected based on rivaroxaban dosing recommendations being dependent on renal function, a higher proportion received an initial daily dose of 15 mg (Table S5). Differences in baseline characteristics between the 105 patients with major bleeding and the 4022 patients without major bleeding, who were included in the model population, were similar to the differences between patients with and without major bleeding in the overall XANTUS population (Table S6). The results of the multivariate analysis, which included all factors that showed a tendency to be different in the univariate comparison and those included in the model that were based on medical judgment (Table S2), identified 6 independent factors associated with major bleeding on treatment with rivaroxaban (Figure 2):

1. Concomitant antiplatelet, NSAID, or paracetamol treatment at any time during the study (HR=1.80; 95% CI 1.24–2.61).
2. Uncontrolled hypertension, defined as blood pressure >160/90 mm Hg at baseline (HR=1.79; 95% CI 1.05–3.05).
3. Heavy (>80 g alcohol/d: HR=2.37; 95% CI 1.24–4.53; *P*=0.009) but not moderate (40–80 g alcohol/d: HR=0.96; 95% CI 0.62–1.46; *P*=0.819) alcohol use at baseline.
4. Increasing age at baseline (HR after parameter‐wise shrinkage=1.25 [per 5‐year increase]; 95% CI 1.12–1.38).
5. Heart failure at baseline (HR=1.97; 95% CI 1.36–2.86).
6. Vascular disease at baseline (HR=1.91; 95% CI 1.32–2.77).

These risk factors were confirmed in a sensitivity analysis using major bleeding or death as outcome parameters (Table S7), with the exception of heavy alcohol use (defined in Table S8) and uncontrolled hypertension (defined in Table S1). In the univariate analyses, HRs for concomitant antiplatelets, NSAIDs, and paracetamol were 1.69 (95% CI 1.13–2.55; *P*=0.012), 1.73 (95% CI 0.76–3.92; *P*=0.191), and 2.64 (95% CI 1.34–5.19; *P*=0.005), respectively (Table S9). An additional sensitivity analysis, which included 87% of the overall XANTUS population by imputing missing CrCl values, showed consistent outcomes in the multivariable model (Table S10).

**Figure 3:**
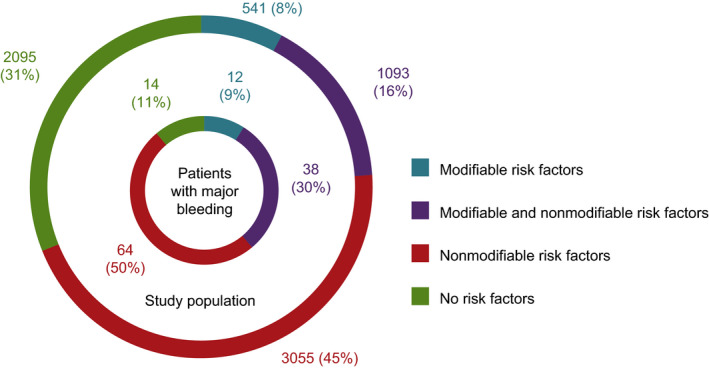
Patient risk profiles. Donut chart showing study population (outer ring) and patients who experienced a major bleeding event (inner ring) according to the presence of bleeding risk factors, split into modifiable and nonmodifiable risk factors. Age ≥75 years was used as a cut‐off point for age to qualify as a nonmodifiable risk factor.

In total, 1634 patients (24%) had at least 1 modifiable bleeding risk factor: 541 had modifiable risk factors only and 1093 patients had both modifiable and nonmodifiable risk factors. Incidence rates of major bleeding (events per 100 patient‐years [95% CI]) increased with the number of modifiable risk factors (0, 1, or 2 risk factors, respectively) from 1.66 (1.31–2.07, no risk factor) to 3.56 (2.62–4.72, 1 risk factor) and 4.16 (0.50–15.02, 2 risk factors) (Table 3). Incidences of major bleeding or death also increased with the number of modifiable risk factors (Table S11). Importantly, 50/128 (39%) patients who had a bleeding event had at least 1 modifiable bleeding risk factor (most of whom also had additional nonmodifiable risk factors) (Figure 3), suggesting that a substantial proportion of bleeding events could be attributable to modifiable bleeding risk factors. An additional analysis testing for interactions between the modifiable risk factors and age (as a continuous risk factor) showed no significant interaction. *P* values for interactions between age and alcohol consumption; concomitant antiplatelet, NSAIDs, or paracetamol use; and uncontrolled hypertension were 0.746, 0.997, and 0.171, respectively (Table S12).

**Table 3:** Major Bleeding in the XANTUS Population Stratified by Number of Modifiable Bleeding Risk Factors

Number of Modifiable Risk Factors	Overall, n (%)	Patients With Major Bleeding^a^, n (%)	Incidence Proportion, % (95% CI)	Incidence Rate Events Per 100 Years (95% CI)
0	5150 (75.9)	78 (1.5)	1.51 (1.20–1.89)	1.66 (1.31–2.07)
1	1577 (23.2)	48 (3.0)	3.04 (2.25–4.02)	3.56 (2.62–4.72)
≥2^b^	57 (0.8)	2 (3.5)	3.51 (0.43–12.11)	4.15 (0.50–14.98)

Number of patients and major bleeding events from the XANTUS (Xarelto for Prevention of Stroke in Patients With Atrial Fibrillation) study were stratified according to number of modifiable risk factors.

a Treatment emergent adjudicated.

b Only 1 patient had 3 modifiable bleeding risk factors and did not experience a bleeding event.

**Figure 4:**
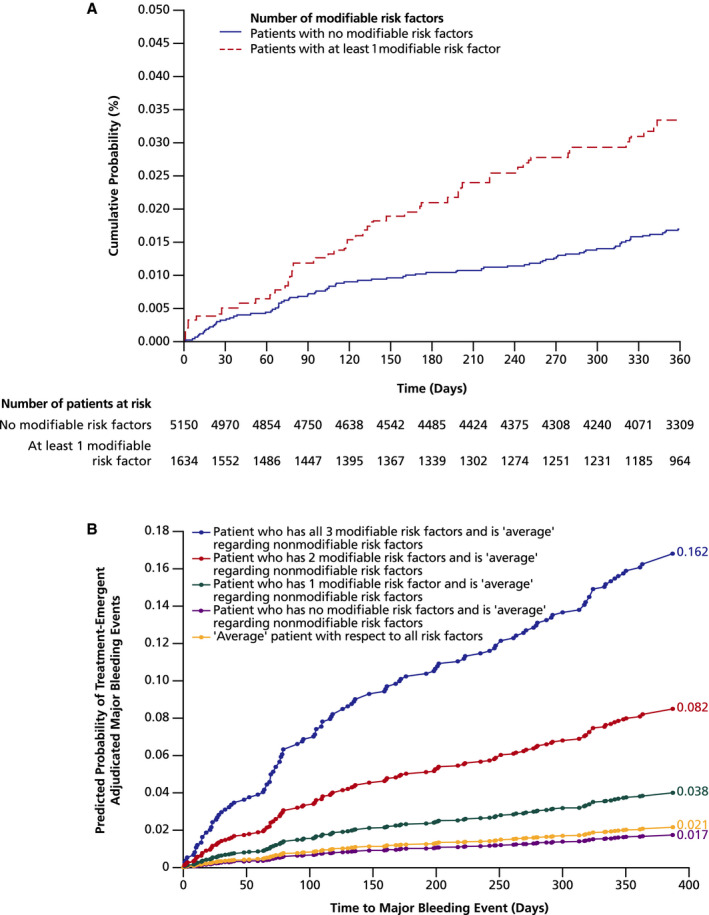
Analysis of bleeding risk in patients with 0 or ≥1 modifiable bleeding risk factors. **A**, Kaplan–Meier curve of bleeding events in patients with 0 or ≥1 modifiable risk factors for bleeding. Patients with ≥1 modifiable risk factor (heavy alcohol use, uncontrolled hypertension, and concomitant therapy with antiplatelet agents, NSAIDs, or paracetamol) were twice as likely to experience a bleeding event compared with patients without modifiable risk factors. **B**, Model‐predicted probabilities of bleeding events in patients with an average profile with respect to all risk factors (yellow), average with respect to all nonmodifiable risk factors and no (purple), 1 (green), 2 (red), or all 3 (blue) modifiable risk factors. Predicted probabilities shown at the end of each projection are for day 360. NSAID indicates nonsteroidal anti‐inflammatory drug.

Patients without modifiable bleeding risk factors had a bleeding risk of 1.7% at day 360, increasing to 3.3% in patients with at least 1 modifiable bleeding risk factor (Figure 4A). The discrimination power of modifiable bleeding risk factors was similar to the discriminatory power of the published HAS‐BLED and ORBIT bleeding risk scores (Table S13): major bleeding incidence rates (events per 100 patient‐years [95% CI]) for patients with low, medium, and high HAS‐BLED bleeding risk (score=0, 1–2, and ≥3, respectively) were 0.36 (0.01–2.03), 1.89 (1.49–2.35), and 2.88 (2.12–3.82), respectively. Bleeding rates for low, medium, and high ORBIT score (0–2, 3, and 4–7, respectively) were 2.61 (1.72–3.80), 5.89 (3.13–10.07), and 5.54 (2.76–9.91), respectively.

The increased bleeding risk was confirmed in a modeling analysis estimating the impact of modifiable bleeding risk factors in patients with an average profile of nonmodifiable risk factors. The probability of major bleeding events rose from 2% at day 360 in patients without modifiable bleeding risk factors to 16% in patients with all 3 modifiable risk factors (Figure 4B). The partial population‐attributable risk analysis showed that eliminating all modifiable bleeding risk factors would reduce the bleeding risk by up to 16%; values attributable to bleeding risk for each modifiable risk factor can be found in Table 4.

**Table 4:** Attributable Bleeding Risk for Modifiable Risk Factors in XANTUS

Risk Factors	Partial PAR (95% CI)
Uncontrolled hypertension	0.025 (−0.019 to 0.069)
Heavy alcohol use	0.017 (−0.008 to 0.042)
Concomitant antiplatelets, NSAIDs, or paracetamol use	0.126 (0.008 to 0.242)
Uncontrolled hypertension and heavy alcohol use	0.042 (−0.020 to 0.104)
Uncontrolled hypertension and concomitant antiplatelets, NSAIDs, or paracetamol use	0.149 (0.004 to 0.289)
Heavy alcohol use and concomitant antiplatelets, NSAIDs, or paracetamol use	0.140 (0.014 to 0.262)
Uncontrolled hypertension and heavy alcohol use and concomitant antiplatelets, NSAIDs, or paracetamol use	0.163 (0.009 to 0.310)

The PAR describes the maximum proportion of major bleeding events that could theoretically be prevented if a specific risk factor and any associated pathologies were to be completely eliminated/reversed from a target population. The partial PAR is of interest where there is more than 1 risk factor of interest and when the set of risk factors includes modifiable and nonmodifiable risk factors. NSAID indicates nonsteroidal anti‐inflammatory drug; PAR, population‐attributable risk; XANTUS, Xarelto for Prevention of Stroke in Patients With Atrial Fibrillation.

## Discussion

### Main Findings

This analysis quantified the impact of modifiable and nonmodifiable bleeding risk factors in an unselected cohort of patients with AF treated with rivaroxaban in routine clinical practice. Iterative analyses identified 3 modifiable and 3 nonmodifiable risk factors associated with bleeding in patients with AF receiving rivaroxaban. Almost 40% of major bleeding events occurred in patients with at least 1 modifiable risk factor. Elimination of modifiable bleeding risk factors (eg, heavy alcohol use, uncontrolled hypertension, and concomitant therapy with antiplatelet agents, NSAIDs, or paracetamol) and in the context of integrated AF care^14^, ^15^ thus emerges as a potentially effective intervention to reduce bleeding risk in anticoagulated patients with AF.

### Modifiable Bleeding Risk Factors in Anticoagulated Patients With AF

The presence of 1 or more of the 3 independent modifiable bleeding risk factors identified in this analysis approximately doubled the risk of major bleeding (Figure 4A); the modeling analysis showed additive effects with an increasing number of risk factors (Figure 4B). Thirty‐nine percent of major bleeding events occurred in patients with at least 1 modifiable risk factor, suggesting that it may have been possible to reduce the risk of bleeding in a large proportion of the study population. An additional benefit of a reduced risk of bleeding is a potentially improved treatment adherence and persistence, which could lead to preventing thromboembolic events more effectively. Furthermore, reducing life‐threatening bleeding events may be an important factor in reducing the risk of overall mortality in patients with AF receiving NOACs.

Concomitant therapy with antiplatelet drugs or NSAIDs increased bleeding risk in our study, confirming observations made by others.^6^, ^8^ NSAIDs reduce gastric protection and inhibit cyclooxygenase in platelets.^16^ The mechanism of action of paracetamol is less clear, but it is thought to involve the inhibition of cyclooxygenase and may also reduce gastric protection, although it is generally considered to be a safer alternative to NSAIDs in patients at an increased risk of bleeding.^17^ In XANTUS, there was no difference in bleeding risk in patients on paracetamol compared with those on NSAIDs. The increased risk of bleeding associated with paracetamol use may be surprising, but it is consistent with another recent analysis.^18^ It was not possible to determine conclusively whether this resulted directly from the use of paracetamol or another associated factor. Paracetamol use did not correlate with any other risk factors included in the analysis, but it is possible that the association could have resulted from the preferential prescription of paracetamol to patients who were already at an increased risk of bleeding.^17^ Overall, these results underpin the need to avoid antiplatelet or NSAID exposure in anticoagulated patients with AF,^3^ which are still commonly coprescribed.^19^

Additional analyses demonstrated that there were no differences in bleeding risk when antiplatelet agents, NSAIDs, and paracetamol were analyzed separately (Table S9), suggesting that the bleeding risk in patients receiving antiplatelet agents, which are usually taken chronically, and paracetamol/NSAIDs, which are normally taken on an as‐needed, short‐term basis, are similar. One possible explanation for this is that chronic pain management in elderly patients with AF (eg, due to arthritis or arthrosis) can result in regular use of paracetamol/NSAIDs. It should be noted that cessation of antiplatelet therapy to reduce the risk of bleeding may not be clinically indicated in all patients with AF, such as those with a recent acute coronary syndrome or recent elective percutaneous coronary intervention, for whom guidelines suggest concomitant antiplatelet and anticoagulation therapy for a limited 6‐ to 12‐month time period.^3^

Uncontrolled hypertension is an independent risk factor for intracranial hemorrhage in anticoagulated^5^, ^6^, ^7^ and nonanticoagulated^20^, ^21^, ^22^ patients, consistent with our analysis. Elevated pulse pressure and intravascular pressure driving chronic arterial vessel damage and precipitating acute rupture of vulnerable vessels are potential explanations for this association. In view of the additional benefits of controlling hypertension for stroke prevention,^20^ optimal control of blood pressure should be a priority in anticoagulated patients with AF at risk of stroke.

The link between heavy alcohol consumption and bleeding is less well understood. Gastritis, esophagitis or liver dysfunction, and alcohol‐induced inhibition of platelets^23^, ^24^ could be responsible for this effect, in addition to the potentially increased chance of overdosing.

### Comparison to Published Bleeding Risk Scores

Concomitant antiplatelet therapy is a component of the HAS‐BLED and ORBIT scores; heavy alcohol use is included in HAS‐BLED and HEMORR_2_HAGES; and hypertension or uncontrolled hypertension are included in the ATRIA, HAS‐BLED, and HEMORR_2_HAGES scores. These scores were developed and validated in patients treated with vitamin K antagonists or in randomized trials evaluating NOAC therapy (AMADEUS [Evaluating the Use of SR34006 Compared to Warfarin or Acenocoumarol in Patients With Atrial Fibrillation], ARISTOTLE [Apixaban for Reduction in Stroke and Other Thromboembolic Events in Atrial Fibrillation], and RE‐LY [Randomized Evaluation of Long Term Anticoagulant Therapy]).^5^, ^6^, ^7^, ^8^, ^9^ In addition, the HAS‐BLED, ATRIA, and ORBIT scores have been compared using registry data on patients receiving NOACs and were found to have modest predictive value.^25^ Our analysis confirmed these bleeding risk factors in an unselected cohort of patients treated with rivaroxaban in routine clinical practice, and the predictive discrimination of independent risk factors in our analysis was compared with ORBIT and HAS‐BLED scores using Harrell's C analysis. As well as the 3 modifiable bleeding risk factors, 3 additional, nonmodifiable bleeding risk factors were identified (age, vascular disease, and heart failure).^3^ Age is a component of the ATRIA, HAS‐BLED, HEMORR_2_HAGES, and ORBIT bleeding risk scores. Vascular disease is not included in these scores and emerges as a novel bleeding risk factor in this analysis. The finding that heart failure is a risk factor for major bleeding in the XANTUS population is in accordance with another large retrospective study of unselected patients with AF treated with rivaroxaban in routine clinical practice.^26^ All 3 are recognized stroke risk factors and part of the CHA_2_DS_2_‐VASc score. Although potentially useful to identify patients who are at high risk of stroke and bleeding, these factors will not inform the decision for anticoagulation.^3^, ^4^, ^19^

Major bleeding events were found in ≈2% of patients per year of treatment in this analysis, which is within the range of the rates of major bleeding observed in the phase III NOAC trials.^27^, ^28^, ^29^, ^30^ The bleeding rate appears acceptable compared with the stroke risk in similar patients without anticoagulation,^3^, ^31^ but this also underpins the need to reduce bleeding risk further in patients with AF receiving NOAC therapy.

Several additional risk factors for bleeding in anticoagulated patients with AF have been proposed,^5^, ^6^, ^7^, ^8^, ^9^ such as anemia, chronic kidney disease, impaired liver function, history of major bleeding, or previous stroke. No association between these risk factors and major bleeding was found in XANTUS, suggesting these are less relevant in patients treated with rivaroxaban.

### Limitations

Enrollment into XANTUS was based on voluntary participation by centers and patients, which may have created patient or physician selection bias. Predefined criteria for events and central adjudication are means to ensure internal validity of the results, but replication in independent cohorts is warranted. Several biomarkers are associated with bleeding events in patients on anticoagulation treatment,^7^, ^9^ but biomarkers were not recorded in XANTUS and, therefore, their effect on bleeding cannot be measured. Patient risk factors for bleeding events, except for CrCl, weight, and concomitant medications, were based on patient characteristics recorded at the screening visit before enrollment into the study; potential changes in patient risk factors (eg, development of hypertension or changes in alcohol consumption) over the duration of the study were not considered. Additionally, patient‐reported measures were used to estimate alcohol consumption; it is possible that some patients did not estimate their consumption accurately. Some heavy drinkers may have been included in the missing data group and therefore excluded from the analysis. Moreover, because of the noninterventional study design, a large proportion (39%) of patients had missing data and were excluded from the multivariate model selection procedure, which may limit the validity of the data set; however, an additional sensitivity analysis, imputing missing CrCl values (the most frequent source of missing data), showed consistent outcomes in the multivariable model. Additionally, because this analysis aimed to identify a simple model of risk factors, potential interactions between risk factors were not considered in the model selection. Nonetheless, interactions between age and modifiable risk factors, which were assessed by adding these variables individually to the final model, were not significant. The study only included patients receiving rivaroxaban and it is therefore unclear whether the findings also apply to other NOACs. Lastly, the potential impact of modifying bleeding risk factors to reduce bleeding risk assumes complete elimination and reversal of any pathological changes associated with said risk factor (eg, liver disease caused by heavy alcohol consumption or peptic ulcers causally associated with use of antiplatelet agents/NSAIDs); therefore, this represents a maximal theoretical benefit and would ideally be confirmed in a controlled trial comparing interventions to reduce modifiable bleeding risk factors with usual care. Without such data, it seems prudent to control blood pressure, to avoid heavy alcohol consumption, and reduce exposure to antiplatelets, NSAIDs, and paracetamol (unless clinically indicated) to minimize bleeding events in patients with AF anticoagulated with rivaroxaban.

## Conclusions

This analysis identified 3 modifiable factors that increase bleeding risk in patients receiving NOAC therapy with rivaroxaban; elimination or reduction of these risk factors may reduce major bleeding events in anticoagulated patients with AF.

## Sources of Funding

This work was supported by Bayer AG (Leverkusen, Germany) and Janssen Research & Development, LLC (Raritan, NJ).

## Disclosures

Kirchhof has received research support from the European Union, the British Heart Foundation (London, UK), the Leducq Foundation (Paris, France), the German Centre for Cardiovascular Research (DZHK, Berlin, Germany), and from several drug and device companies active in atrial fibrillation (significant); he has also received honoraria from several such companies, including Bayer, Boehringer Ingelheim, Pfizer/Bristol‐Myers Squibb, and Daiichi Sankyo (modest). He is listed as an inventor on 2 pending patents held by the University of Birmingham. Haas has served as a consultant for Aspen, Bayer, Bristol‐Myers Squibb, Daiichi Sankyo, Pfizer, and Sanofi (modest). Amarenco has served as a consultant for Bayer (significant), Bristol‐Myers Squibb (modest), Pfizer (modest), Boehringer Ingelheim (modest), Daiichi Sankyo (modest), AstraZeneca (significant), Sanofi (modest), Boston Scientific (modest), Edwards (modest), Lundbeck (modest), Merck (modest), and Kowa Pharmaceutical (modest). Hess and van Eickels are employees of Bayer AG (significant). Lambelet is an employee of Chrestos Concept, which received funding for this analysis from Bayer AG (modest). Turpie has been a consultant for Bayer, Janssen Pharmaceutical Research & Development LLC, Astellas, Portola, and Takeda (significant). Camm has received institutional research grants and personal fees as an advisor or speaker from Bayer (significant), Boehringer Ingelheim (modest), Pfizer/Bristol‐Myers Squibb (modest), and Daiichi Sankyo (modest).

## Supporting information


**Appendix S1.** XANTUS Investigators.
**Table S1.** Definitions of Uncontrolled Hypertension, Vascular Disease, and Heart Failure
**Table S2.** Risk Factors Selected After Univariate Analysis for Inclusion in the Multivariable Cox Regression Model
**Table S3.** Internal Validation of XANTUS Bleeding Model Using an Optimism‐Corrected Version of Harrell's C‐Index
**Table S4.** Baseline Demographics and Clinical Characteristics of Patients With and Without Treatment‐Emergent Major Bleeding Events or Treatment‐Emergent Death Events in XANTUS
**Table S5.** Baseline Demographics and Clinical Characteristics of Patients Included in, and Excluded From, the Multivariable Model Selection Procedure
**Table S6.** Baseline Demographics and Clinical Characteristics of Patients With and Without Treatment Emergent Major Bleeding in the Model Population (n=4127)
**Table S7.** Independent Factors Associated With Major Bleeding or Death in the XANTUS Population (N=6784)
**Table S8.** Alcohol Equivalence
**Table S9.** Antithrombotic Treatment as Risk Factors for Major Bleeding
**Table S10.** Independent Factors Associated With Major Bleeding in the XANTUS Population in a Sensitivity Analysis With Imputation of Missing CrCl Values (n=5896)
**Table S11.** Major Bleeding or Death in the XANTUS Population Stratified by the Number of Modifiable Bleeding Risk Factors
**Table S12.** Assessment of Interactions Between Age and Modifiable Risk Factors
**Table S13.** Major Bleeding in the XANTUS Population Stratified by HAS‐BLED and ORBIT Bleeding Risk Scores and Validation of the XANTUS Bleeding Score Using Harrell's C‐Index
**Figure S1.** Graphical assessment of the proportional hazards assumption of the risk factors.
**Figure S2.** Assessment of linearity for age—smoothed plot of Martingale residuals.
**Figure S3.** Calibration plot assessing correlation between actual and predicted probabilities in the final multivariate model.
