# Supplementary material for: Impact of Modifiable Bleeding Risk Factors on Major Bleeding in Patients With Atrial Fibrillation Anticoagulated With Rivaroxaban
Source: J Am Heart Assoc. 2020 Feb 21;9(5):e009530. doi: 10.1161/JAHA.118.009530 (PMC7335544; doi:10.1161/JAHA.118.009530)
Supplement: Supplementary file 1 — Appendix S1. XANTUS Investigators. Table S1. Definitions of Uncontrolled Hypertension, Vascular Disease, and Heart Failure Table S2. Risk Factors Selected After Univariate Analysis for Inclusion in the Multivariable Cox Regression Model Table S3. Internal Validation of XANTUS Bleeding Model Using an Optimism‐Corrected Version of Harrell's C‐Index Table S4. Baseline Demographics and Clinical Characteristics of Patients With and Without Treatment‐Emergent Major Bleeding Events or Treatment‐Emergent Death Events in XANTUS Table S5. Baseline Demographics and Clinical Characteristics of Patients Included in, and Excluded From, the Multivariable Model Selection Procedure Table S6. Baseline Demographics and Clinical Characteristics of Patients With and Without Treatment Emergent Major Bleeding in the Model Population (n=4127) Table S7. Independent Factors Associated With Major Bleeding or Death in the XANTUS Population (N=6784) Table S8. Alcohol Equivalence Table S9. Antithrombotic Treatment as Risk Factors for Major Bleeding Table S10. Independent Factors Associated With Major Bleeding in the XANTUS Population in a Sensitivity Analysis With Imputation of Missing CrCl Values (n=5896) Table S11. Major Bleeding or Death in the XANTUS Population Stratified by the Number of Modifiable Bleeding Risk Factors Table S12. Assessment of Interactions Between Age and Modifiable Risk Factors Table S13. Major Bleeding in the XANTUS Population Stratified by HAS‐BLED and ORBIT Bleeding Risk Scores and Validation of the XANTUS Bleeding Score Using Harrell's C‐Index Figure S1. Graphical assessment of the proportional hazards assumption of the risk factors. Figure S2. Assessment of linearity for age—smoothed plot of Martingale residuals. Figure S3. Calibration plot assessing correlation between actual and predicted probabilities in the final multivariate model. [file JAH3-9-e009530-s001.pdf]

SUPPLEMENTAL MATERIAL

Appendix

XANTUS Investigators

Germany: A. Al-Zoebi, N. Schön, O. Scheuermann, T. Schröder, G. Weyers, M. Salefsky, K.-F. Appel, K. Jocham, W. Rieker, A. Schnabel, R. Bosch, J. Dahm, J. Brachmann, C. Schwencke, S. Ragab, W. Jung, G. Stenzel, M. Antz, M. Grebe, H. Diedrichs, A. Mügge, M. Gabelmann, U. Hoffmann, A. Krummenerl, M. Leicht, J. Merke, N. Proskynitopoulos, F. Richard, U. Schulze, J. Taggeselle, M. Conze, U. Frees, M. Leschke, H. Heuer, C. Piper, H.-E. Sarnighausen, A. Krämer, G. Hübner, P. Krings, C. Stellbrink, C. Bauknecht, G. Nickenig, B. Hügl, R. Steinhard, R. Hambrecht, M. Kroll, D. Gulba, R. Stöhring, L. Drude, A. Stadelmann, P. Schwimmbeck, C. Axthelm, F. Pröpper; **United Kingdom:** C. Harinath, C. Kausik, J. Finlayson, A. Dixit, F. Osman, P. Davey, D. Mangion, P. Nair, H. Kadr, P. Sharma, R. Arya, P. Kirchhof; **France:** P. Amarenco, M. Pillot, S. Ansaldi, J.-P.Bellefleur, K. Bouchlaghem, P. Chemin, P. Chevalier, C. Chisseray Pramotton, R. Chokron, A. Ciausu, D. Coisne, S. Combes, Y. Cottin, R. Crespy, A. Dabboura, P. Dematteo, M. Elbaz, J.-L. Farges, J.-M. Faucheux, L. Fauchier, E. Foltzer, P.-Y. Fournier, J. Gauthier, B. Grivet, B. Guillon, R. Khalvadjian, B. Koujan, J.-N. Labeque, J.-P. Labbe, J.-L. Long, M. Loubet, P. Samama, M. Martelet, S. Mielot, J.-P. Neau, V. Probst, A. Rifai, Y. Samson, B. Truong Minh Ky, F. Viader, A. Corbin, P. Dafaye, P. Lang, A. Dompnier, A. Hagege, N. Lellouche, S. Destrac, N. Nighoghossian, S. Guerard, J. M. Davy, J. F. Aupetit, A. Durand Dubief, A. Gacem, M. Zylberberg, E. Fournier, S. Kownator, W. Amara, J. P. Elkaim, E. Ferrari, G. Napporn, P. F. Winum, E. Aliot, M. Sarov-Riviére; **Poland:** Z. Kornacewicz-Jach, E. Zinka, M. Nartowicz-Słoniewska, B. Mocarska-Górna, G. Latocha-Korecka, A. Stasiewski, J. Tyszkiewicz, T. Lepich, R. Kluba, W. Piotrowski, B. Mikłaszewicz, M. Pawłowska, S. Mazur; **Canada:** G. Houde, F. Grondin, B. Craig, C. Lai, S. Lam, A. Kucktaruk, G. Sabe-Affaki, M. Miller, L. Hill, A. Kelly, S. Fikry, F. St-Maurice, P. Filteau, A. Fréchette, M. Csanadi, M. Mason, G. Chouinard, R. Gendreau; **Belgium:** P. Melon, L. De Wolf, H. Heidbüchel, L. Janssens, C. Scavée, E. Hoffer, J. Leroy,

T. Boussy; **Netherlands:** A. Elvan, W. Hermans, H. Suryapranata, M. van Eck, P. Van Haelst, J. Meeder, P. Hoogslag, H. Kragten, R. Tukkie, M. Hemels, L. Van Wijk, P. Nierop, W. Ten Holt, P. Zwart, A. Bakx, A. Kuijper, R. de Nooijer, R. Van der Heijden, J. ten Berg, H. Beyerbacht, J. L. Brouwer, B. Van den Berg, F. Den Hartog; **Sweden:** C. Blomström Lundqvist, A.-C. Laska, C. Rorsman, E. Moor, C.-J. Lindholm, S. Lind, J. Teichert, T. Grähs, L. Falk, S. Bandh; **Denmark:** H. Nielsen, S. Riahi, S. Pehrson, E. Zeuten, G. Jensen, G. Gislason, H. Ibsen, P. Hildebrandt; **Norway:** E. Øie, M. Kurz, P. K. Rønnevik; **Czech Republic:** O. Škoda, F. Málek, M. Souček, J. Spinar, D. Marek, R. Mikulik, P. Jansky, M. Bar, M. Barsovsky, L. Krajcova, M. Homza; **Israel:** R. Shohat Zabarski, W. Nseir, Z. Vered, U. Rosenschein; **Portugal:** J. Martinez, P. Canhão, T. Gregorio, R. Fontes Carvalho; **Austria:** H. J. Nesser, U. Hoppe, B. M. Pieske, J. R. Weber, C. Brenneis, A. Winkler, K. Huber, A. Gatterer, A. Podczeck-Schweighofer, M. Gwechenberger, F. Weidinger; **Hungary:** Z. Csanadi, Z. Jarai, A. Vertes, K. Zamolyi; **Russia:** A. Tarasov, I. Baychorov, M. Kubova, M. Ryabinina, S. Matskeplishvilli, V. Sulimov, S. Kozhevnikova, E. Adonina, V. Lartseva, A. Shimkevich, A. Rychkov, N. Khorkova, O. Kolycheva, S. Zenin, O. Kononenko, E. Vlasyants, M. Dereva, E. Vedeneva, E. Minasova, S. Popov, S. Usenkov, I. Kisteneva, R. Batalov, G. Spacenkov, S. Kuznetsov, T. Tsybulskaya, M. Vitsenya, O. Vyborov, I. Shlyk, I. Voznyuk, A. Muhamedrahimova, A. Obrezan; **Slovakia:** M. Tvrdikova, S. Filipova, E. Roskova, J. Strbova, S. Farsky, D. Pella, I. Nedělová, F. Fazekas, J. Pacherová, Z. Lašanová, P. Hlivák, P. Spurný, V. Pokorna, M. Mora, M. Stefanik; **Ukraine:** O. Karlinska, O. Sychov, T. Grygorieva, O. Gukov, O. Romanenko, T. Petrovskyy, M. Gorbach, D. Reshotko, G. Maxym; **Slovenia:** V. Kanic, M. Marija, G. Mirjam, T. Šubic; **Ireland:** N. Mahon; **Moldova:** A. Grosu, A. Grivenco, R. Grajdieru, N. Diaconu.

Table S1. Definitions of uncontrolled hypertension, vascular disease, and heart failure.

Parameter	Definition used
Uncontrolled hypertension	Blood pressure >160/90 mm Hg
Vascular disease	Recorded according to type (peripheral artery disease, ischemic heart disease, or cerebrovascular disease), as assessed by the treating physician
Heart failure	Clinically defined using reduced left ventricular ejection fraction

Table S2. **Risk factors selected after univariate analysis for inclusion in the multivariable Cox regression model.**

	HR (95% CI)	*P*-value
**Risk factors included in multivariate model based on *P*<0.1 in univariate analysis**		
Age at baseline (5-year increase)	1.31 (1.18–1.45)	<0.001
Heart failure at baseline: Yes vs No	2.42 (1.68–3.47)	<0.001
Vascular disease at baseline: Yes vs No	2.31 (1.63–3.28)	<0.001
Concomitant antiplatelet therapy, NSAIDs, or paracetamol use at any time during study: Yes vs No	1.97 (1.36–2.86)	<0.001
Rivaroxaban dose (first documented): Overall		<0.001
20 mg vs 15 mg	0.57 (0.39–0.83)	0.003
Other or missing vs 15 mg	3.16 (0.98–10.23)	0.055
History of hypertension: Yes vs No	1.80 (1.12–2.91)	0.015
Concomitant dual antiplatelet therapy at any time during study: Yes vs No	2.99 (1.22–7.32)	0.016
Hepatic insufficiency: Yes vs No	2.38 (1.05–5.40)	0.038
Alcohol consumption at baseline: Overall		0.091
Alcohol consumption at baseline: Heavy vs Abstinent or mild	3.12 (0.99–9.82)	0.052
Alcohol consumption at baseline: Medium vs Abstinent or mild	0.71 (0.35–1.46)	0.355
**Risk factors included in multivariate model based on medical judgment**		
Concomitant CYP3A4 or P-gp inhibitors* at any time during study (modelled as time dependent): Yes vs No	1.41 (0.93–2.13)	0.105
Renal insufficiency: First available CrCl <50 mL/min vs ≥50 mL/min	1.46 (0.92–2.33)	0.109
Uncontrolled hypertension: Yes vs No	1.64 (0.80–3.36)	0.175
Anemia or reduced hemoglobin[#]: Yes vs No	1.70 (0.75–3.85)	0.207
Prior stroke: Yes vs No	1.27 (0.80–2.02)	0.301

Sex: Male vs Female	1.18 (0.82–1.69)	0.368
First available weight: >60 kg vs ≤60 kg	0.86 (0.46–1.61)	0.644
Previous labile INR documented: Yes vs No	1.11 (0.68–1.81)	0.676

*Strong, moderate and weak inhibitors were included.

#Anemia as recorded at baseline; hemoglobin data based on first available data recorded at baseline or at any time during the study.

CI indicates confidence interval; CrCl, creatinine clearance; CYP3A4, cytochrome P450 3A4; HR, hazard ratio; INR, international normalized ratio; NSAID, nonsteroidal anti-inflammatory drug; P-gp, P-glycoprotein.

Table S3. Internal validation of XANTUS bleeding model using an optimism-corrected version of Harrell's C-index.

Apparent C*	Averaged optimism Estimate O#	Harrell's C corrected for optimism estimation†
0.728	0.036	0.692

*Calculation of the apparent C using all patients with backward selection of the variables.

#Average value from 200 bootstrap samples.

†Corrected for optimism estimation.

Table S4. Baseline demographics and clinical characteristics of patients with and without treatment-emergent major bleeding events or treatment-emergent death events in XANTUS.

	All patients (N=6784)	Patients with major bleeding or death (n=232)	Patients without major bleeding or death (n=6552)	*P*-value
Age years, mean ± SD	71.5±9.95	76.6±10.07	71.3±9.89	<0.001
<75 years, n (%)	3975 (58.6)	90 (38.8)	3885 (59.3)	<0.001
≥75 years, n (%)	2809 (41.4)	142 (61.2)	2667 (40.7)	<0.001
Male, n (%)	4016 (59.2)	137 (59.1)	3879 (59.2)	0.9566
Body mass index, kg/m^2, mean ± SD	28.3±4.98	27.6±5.68	28.4±4.95	0.0425
First available creatinine clearance, n (%)				
<80 mL/min	2961 (43.6)	145 (62.5)	2816 (43.0)	0.0049
≥80 mL/min	1491 (22.0)	46 (19.8)	1445 (22.1)	0.0049
Missing	2332 (34.4)	41 (17.7)	2291 (35.0)	0.0049
Hepatic insufficiency, n (%)*	137 (2.0)	12 (5.2)	125 (1.9)	0.0005
Rivaroxaban dose (first documented), n (%)				

15 mg	1410 (20.8)	82 (35.3)	1328 (20.3)	<0.0001
20 mg	5336 (78.7)	144 (62.1)	5192 (79.2)	<0.0001
Other/missing	38 (0.6)	6 (2.6)	32 (0.5)	<0.0001
Concomitant ASA or NSAIDs, n (%)	1118 (16.5)	51 (22.0)	1067 (16.3)	0.0215
Concomitant dual antiplatelets, n (%)	105 (1.5)	8 (3.4)	97 (1.5)	0.0170
Concomitant antiplatelet, NSAIDs, or paracetamol, n (%)	1363 (20.1)	72 (31.0)	1291 (19.9)	<0.0001
Concomitant CYP3A4 or P-gp inhibitors, n (%)[#]	1313 (19.4)	59 (25.4)	1254 (19.1)	0.0171
Concomitant paracetamol, n (%)	191 (2.8)	21 (9.1)	170 (2.6)	<0.0001
Active cancer, n (%)	105 (1.5)	9 (3.9)	96 (1.5)	0.0034
Prior bleeding, n (%)	49 (0.7)	3 (1.3)	46 (0.7)	0.2961
Ulcerative gastrointestinal disease, n (%)	27 (0.4)	2 (0.9)	25 (0.4)	0.2533
Uncontrolled hypertension, n (%)	275 (4.1)	10 (4.3)	265 (4.0)	0.8401
Prior stroke, n (%)	935 (13.8)	48 (20.7)	887 (13.5)	0.0019
Prior MI, n (%)	688 (10.1)	41 (17.7)	647 (9.9)	0.0001
Heart failure at baseline, n (%)	1265 (18.6)	92 (39.7)	1173 (17.9)	<0.0001
Platelet count <80 000, n (%)	39 (0.6)	2 (0.9)	37 (0.6)	0.9282

Diabetes mellitus, n (%)	1333 (19.6)	60 (25.9)	1273 (19.4)	0.0154
Vascular disease, n (%)	1685 (24.8)	91 (39.2)	1594 (24.3)	<0.0001
Heavy alcohol use, n (%)	54 (0.8)	5 (2.2)	49 (0.7)	0.0089
Anemia/reduced hemoglobin, n (%)	203 (3.0)	19 (8.2)	184 (2.8)	<0.0001
Known coagulopathy, n (%)	19 (0.3)	2 (0.9)	17 (0.3)	0.0878
Bridging therapy during interruptions, n (%)	100 (1.5)	12 (5.2)	88 (1.3)	<0.0001

The baseline demographics and clinical characteristics from patients from the XANTUS study were stratified according to the presence or absence of major bleeding or death.

*Defined as ‘abnormal liver function’ by the study investigator.

#Strong, moderate, and weak inhibitors were included.

ASA indicates acetylsalicylic acid; CYP3A4, cytochrome P450 3A4; MI, myocardial infarction; NSAID, nonsteroidal anti-inflammatory drug; P-gp, P-glycoprotein; SD, standard deviation.

Table S5. Baseline demographics and clinical characteristics of patients included in, and excluded from, the multivariable model selection procedure.

	Patients included in multivariate model (n=4127)	Patients excluded from multivariate model because of ≥1 missing value (n=2657)	*P*-value
Age years, mean ± SD	71.6±10.08	71.4±9.73	0.5007
<75 years, n (%)	2402 (58.2)	1573 (59.2)	0.4144
≥75 years, n (%)	1725 (41.8)	1084 (40.8)	
Male, n (%)*	2423 (58.7)	1593 (60.0)	0.2834
BMI, kg/m^2, mean ± SD	28.2±4.98	28.6±4.97	0.0273
First available CrCl, n (%)	–	–	0.0098
<50 mL/min	3518 (85.2)	294 (90.5)	
≥50 mL/min	609 (14.8)	31(9.5)	
Missing	0	2332	
Hepatic insufficiency, n (%)[#]	95 (2.3)	42 (1.6)	0.0393
Rivaroxaban dose (first documented), n (%)	–	–	<0.0001
15 mg	931 (22.6)	479 (18.0)	
20 mg	3174 (76.9)	2162 (81.4)	

Other/missing	22 (0.5)	16 (0.6)	
Concomitant ASA or NSAIDs, n (%)	707 (17.1)	411 (15.5)	0.0716
Concomitant dual antiplatelets, n (%)	66 (1.6)	39 (1.5)	0.6687
Concomitant antiplatelet, NSAIDs, or paracetamol, n (%)	872 (21.1)	491 (18.5)	0.0078
Concomitant CYP3A4 or P-gp inhibitors, n (%)†	855 (20.7)	458 (17.2)	0.0004
Concomitant paracetamol, n (%)	150 (3.6)	41 (1.5)	<0.0001
Active cancer, n (%)	72 (1.7)	33 (1.2)	0.1016
Prior bleeding, n (%)	31 (0.8)	18 (0.7)	0.7264
Ulcerative gastrointestinal disease, n (%)	18 (0.4)	9 (0.3)	0.5339
Uncontrolled hypertension, n (%)	149 (3.6)	126 (4.7)	0.0210
Prior stroke, n (%)‡	608 (14.7)	327 (12.3)	0.0049
Prior MI, n (%)	440 (10.7)	248 (9.3)	0.0770
Heart failure at baseline, n (%)	851 (20.6)	414 (15.6)	<0.0001
Platelet count, n (%)	–	–	<0.0001
<80 000	23 (0.9)	16 (4.8)	
≥80,000	2529 (99.1)	315 (95.2)	
Missing	1575	2326	

Diabetes mellitus, n (%)	856 (20.7)	477 (18.0)	0.0048
Vascular disease, n (%)	1097 (26.6)	588 (22.1)	<0.0001
Alcohol consumption, n (%)	–	–	0.5844
Abstinent or mild	3724 (90.2)	2209 (89.6)	
Medium	372 (9.0)	234 (9.5)	
Heavy	31 (0.8)	23 (0.9)	
Missing	0	191	
Anemia/reduced hemoglobin, n (%)	162 (3.9)	41 (1.5)	<0.0001
Known coagulopathy, n (%)	9 (0.2)	10 (0.4)	0.2285
Bridging therapy during interruptions, n (%)	80 (1.9)	20 (0.8)	<0.0001

% and *P*-values are based on number of patients with available data for each characteristic.

*Information missing for 3 patients excluded from the multivariate model.

[#]Defined as ‘abnormal liver function’ by the study investigator.

[†]Strong, moderate and weak inhibitors were included.

[‡]Information missing for 2 patients excluded from the multivariate model.

ASA indicates acetylsalicylic acid; BMI, body mass index; CrCl, creatinine clearance; CYP3A4, cytochrome P450 3A4; MI, myocardial infarction; NSAID, nonsteroidal anti-inflammatory drug; P-gp, P-glycoprotein; SD, standard deviation.

Table S6. Baseline demographics and clinical characteristics of patients with and without treatment emergent major bleeding in the model population (n=4127).

	Patients with major bleeding (n=105)	Patients without major bleeding (n=4022)	*P*-value
Age years, mean ± SD	75.9±9.5	71.5±10.1	<0.0001
<75 years, n (%)	42 (40.0)	2360 (58.7)	0.0001
≥75 years, n (%)	63 (60.0)	1662 (41.3)	
Male, n (%)	64 (61.0)	2359 (58.7)	0.6365
BMI, kg/m^2, mean ± SD	28.33±5.41	28.23±4.97	0.8544
First available CrCl, n (%)			0.0698
<50 mL/min	22 (21.0)	587 (14.6)	
≥50 mL/min	83 (79.0)	3435 (85.4)	
Hepatic insufficiency, n (%)*	6 (5.7)	89 (2.2)	0.0182
Rivaroxaban dose (first documented), n (%)			0.0033
15 mg	35 (33.3)	896 (22.3)	
20 mg	68 (64.8)	3106 (77.2)	
Other/missing	2 (1.9)	20 (0.5)	

Concomitant ASA or NSAIDs, n (%)	31 (29.5)	676 (16.8)	0.0006
Concomitant dual antiplatelets, n (%)	5 (4.8)	61 (1.5)	0.0089
Concomitant antiplatelet, NSAIDs, or paracetamol, n (%)	38 (36.2)	834 (20.7)	0.0001
Concomitant CYP3A4 or P-gp inhibitors, n (%)[#]	31 (29.5)	824 (20.5)	0.0241
Concomitant paracetamol, n (%)	9 (8.6)	141 (3.5)	0.0062
Active cancer, n (%)	3 (2.9)	69 (1.7)	0.3778
Prior bleeding, n (%)	1 (1.0)	30 (0.7)	0.8088
Ulcerative gastrointestinal disease, n (%)	0 (0)	18 (0.4)	0.4921
Uncontrolled hypertension, n (%)	7 (6.7)	142 (3.5)	0.0890
Prior stroke, n (%)	21 (20.0)	587 (14.6)	0.1229
Prior MI, n (%)	15 (14.3)	425 (10.6)	0.2229
Heart failure at baseline, n (%)	40 (38.1)	811 (20.2)	<0.0001
Platelet count <80 000, n (%)	2 (2.6)	21 (0.8)	0.1051
Diabetes mellitus, n (%)	28 (26.7)	828 (20.6)	0.1293
Vascular disease, n (%)	50 (47.6)	1047 (26.0)	<0.0001
Alcohol consumption, n (%)			0.0299
Abstinent or mild	95 (90.5%)	3629 (90.2)	

Medium	7 (6.7)	365 (9.1%)	
Heavy	3 (2.9)	28 (0.7)	
Anemia/reduced hemoglobin, n (%)	6 (5.7)	156 (3.9)	0.3390
Known coagulopathy, n (%)	1 (1.0)	8 (0.2)	0.1023
Bridging therapy during interruptions, n (%)	11 (10.5)	69 (1.7)	<0.0001

*Defined as 'abnormal liver function' by the study investigator.

#Strong, moderate and weak inhibitors were included.

ASA indicates acetylsalicylic acid; BMI, body mass index; CrCl, creatinine clearance; CYP3A4, cytochrome P450 3A4; MI, myocardial infarction; NSAID, nonsteroidal anti-inflammatory drug; P-gp, P-glycoprotein; SD, standard deviation.

Table S7. Independent factors associated with major bleeding or death in the XANTUS population (N=6784).

Risk factors	HR	95% CI	*P*-value
Heart failure at baseline: Yes vs No	2.27	(1.64–3.16)	<0.001
Age (5-year increase)	1.21	(1.10–1.33)	<0.001
Concomitant antiplatelets, NSAIDs, or paracetamol use: Yes vs No	1.76	(1.26–2.47)	<0.001
Rivaroxaban dose (first documented): Overall			0.003
Rivaroxaban dose (first documented): 20 mg vs 15 mg	0.70	(0.49–0.99)	0.043
Rivaroxaban dose (first documented): Other or missing vs 15 mg	3.35	(1.21–9.28)	0.020
Vascular disease: Yes vs No	1.56	(1.13–2.16)	0.007
Hepatic insufficiency: Yes vs No*	2.27	(1.21–4.27)	0.011
Anemia or reduced hemoglobin use: Yes vs No	1.58	(0.92–2.70)	0.095

Multivariable Cox proportional hazard model of time-dependent risk factors for treatment-emergent adjudicated major bleeding or treatment-emergent adjudicated death. This analysis was performed as a sensitivity analysis for the factors predicting major bleeding. All factors with the exception of heavy alcohol use and uncontrolled hypertension were confirmed in the sensitivity analysis. Hepatic insufficiency, a common consequence of long-term heavy alcohol abuse, was associated with major bleeding or death.

*Defined as 'abnormal liver function' by the study investigator.

CI indicates confidence interval; HR, hazard ratio; NSAID, nonsteroidal anti-inflammatory drug.

Table S8. Alcohol equivalence.

Alcohol consumption category	Gram alcohol per day	Country	Glasses per day		
			**Beer** (5% alcohol by volume)	**Wine** (12% alcohol by volume)	**Spirits** (40% alcohol by volume)
Abstinent	0	All countries	0	0	0
Mild	<40	Germany, France, Italy, Japan	<4 glasses at 0.2 L/glass	<2 glasses at 0.2 L/glass	<5 glasses at 2 cL/glass
		United Kingdom	<1.5 pints	<3 glasses at 125 mL/glass	<4 measures at 25 mL/glass
		Russia	<2.5 glasses at 0.33 L/glass	–	<2 glasses at 50 mL/glass
		North America	<2 cans at 12 fluid ounces/can	<2 glasses at 5 fluid ounces/glass	<2 glasses at 1.5 fluid ounces/glass
Moderate	40–80	Germany, France, Italy, Japan	4–8 glasses at 0.2 L/glass	2–4 glasses at 0.2 L/glass	5–10 glasses at 2 cL/glass
		United Kingdom	1.5–3 pints	3–6 glasses at 125 mL/glass	4–8 measures at 25 mL/glass

		Russia	2.5–5 glasses at 0.33 L/glass	–	2–4 glasses at 50 mL/glass
		North America	2–4 cans at 12 fluid ounces/can	2–5 glasses at 5 fluid ounces/glass	2–4 glasses at 1.5 fluid ounces/glass
Heavy	>80	Germany, France, Italy, Japan	>8 glasses at 0.2 L/glass	>4 glasses at 0.2 L/glass	>10 glasses at 2 cL/glass
		United Kingdom	>3 pints	>6 glasses at 125 mL/glass	>8 measures at 25 mL/glass
		Russia	>5 glasses at 0.33 L/glass	–	>4 glasses at 50 mL/glass
		North America	>4 cans at 12 fluid ounces/can	>5 glasses at 5 fluid ounces/glass	>4 glasses at 1.5 fluid ounces/glass

The categories of alcohol consumption (abstinent, mild, moderate, and heavy) were defined by the total daily alcohol content (in grams) consumed by the individual. The categories were created so that subgroup analyses could be performed.

The definitions used are provided in the Table S5. For example: if an individual consumed beer at <800 mL/day (<40 g alcohol/day), he/she was classified as a mild alcohol consumer. However, if the individual consumed 500 mL/day of beer and 250 mL of wine, he/she was classified as a moderate alcohol consumer (25 g alcohol/day of beer + 30 g alcohol/day of wine = 55 g alcohol/day).

If the country where the trial was being conducted is not listed in the table (eg, Sweden), the closest country could be used. For example, investigators in Sweden could use the ‘Germany, France, Italy, Japan’ category.

Table S9. Antithrombotic treatment as risk factors for major bleeding.

Risk factors	HR*	95% CI	*P*-value
Concomitant antiplatelet, NSAID or paracetamol	1.97	1.36–2.86	<0.001
Concomitant antiplatelet	1.69	1.13–2.55	0.012
Concomitant NSAID or paracetamol	2.19	1.23–3.88	0.007
Concomitant paracetamol	2.64	1.34–5.19	0.005
Concomitant antiplatelet or NSAID	1.77	1.20–2.61	0.004
Concomitant NSAID	1.73	0.76–3.92	0.191

*Shown for the comparison of concomitant antithrombotic therapy yes vs no in separate univariate Cox proportional hazard models.

CI indicates confidence interval; HR, hazard ratio; NSAID, nonsteroidal anti-inflammatory drug.

Table S10. Independent factors associated with major bleeding in the XANTUS population in a sensitivity analysis with imputation of missing CrCl values (n=5896).

**Risk factors**	**HR**	**95% CI**	***P*-value**
Age (5-year increase)	1.27	1.14–1.41	<0.001
Heart failure at baseline: Yes vs No	2.08	1.42–3.07	<0.001
Vascular disease: Yes vs No	1.94	1.33–2.84	<0.001
Concomitant antiplatelets, NSAIDs, or paracetamol use: Yes vs No	1.95	1.32–2.88	<0.001
Alcohol consumption at baseline: Overall			0.090
Alcohol consumption at baseline: Heavy vs Abstinent or mild	3.58	1.13–11.34	0.030
Alcohol consumption at baseline: Medium vs Abstinent or mild	0.91	0.42–1.96	0.806

A backwards selection was done using a *P*-value of 0.1 for variables to stay in the model.

Candidate risk factors were used as for the primary analysis shown in Table S2.

Patients with missing CrCl and CrCl ≥50 mL/min were grouped together because a comparison of baseline characteristics and event rates between patients with and without CrCl showed that those with missing data were generally healthier than those with available data, i.e. they have fewer co-morbidities (e.g. hypertension, prior stroke, heart failure and diabetes) and were less likely to have a stroke, bleeding event or die.

CI indicates confidence interval; CrCl, creatinine clearance; HR, hazard ratio; INR, international normalized ratio; NSAID, nonsteroidal anti-inflammatory drug; P-gp, P-glycoprotein.

Table S11. Major bleeding or death in the XANTUS population stratified by the number of modifiable bleeding risk factors.

Number of modifiable risk factors	Overall n (%)	Patients with major bleeding events or death* n (%)	Incidence proportion % (95% CI)	Incidence rate Events per 100 years (95% CI)
0	5150 (75.9)	148 (2.9)	2.87 (2.43–3.37)	3.15 (2.66–3.70)
1	1577 (23.2)	81 (5.1)	5.14 (4.10–6.34)	6.00 (4.77–7.46)
≥2	57 (0.8)	3 (5.3)	5.26 (1.10–14.62)	6.22 (1.28–18.18)

Number of patients and major bleeding events or death from the XANTUS study were stratified according to the number of modifiable risk factors.

*Treatment-emergent adjudicated.

[#]Only one patient had three modifiable bleeding risk factors and did not experience a bleeding event.

CI indicates confidence interval.

Table S12. Assessment of interactions between age and modifiable risk factors.

Modifiable risk factor	Age <75 years		Age ≥75 years		*P*-value (interaction)*
	Patients with major bleeding n/N (%)	Incidence rate Events per 100 patient-years (95% CI)	Patients with major bleeding n/N (%)	Incidence rate Events per 100 patient-years (95% CI)	
Concomitant antiplatelet, NSAID or paracetamol					0.997
Yes	17/757 (2.2)	2.59 (1.51–4.15)	24/606 (4.0)	4.75 (3.04–7.07)	
No	35/3218 (1.1)	1.18 (0.82–1.65)	52/2203 (2.4)	2.63 (1.97–3.45)	
Alcohol consumption					0.746
Abstinent or mild	42/3371 (1.2)	1.37 (0.99–1.85)	67/2562 (2.6)	2.97 (2.30–3.77)	
Medium	4/448 (0.9)	0.99 (0.27–2.54)	4/158 (2.5)	2.81 (0.77–7.20)	
Heavy	2/42 (4.8)	5.74 (0.70–20.75)	1/12 (8.3)	8.38 (0.21–46.68)	
Uncontrolled hypertension					0.171
Yes	4/159 (2.5)	2.92 (0.80–7.48)	4/116 (3.4)	3.96 (1.08–10.14)	
No	48/3816 (1.3)	1.38 (1.02–1.83)	72 /2693(2.7)	3.03 (2.37–3.81)	

*The interaction *P*-values were calculated using the model including age (continuous), heart failure, vascular disease, concomitant antiplatelet therapy, NSAID or paracetamol, alcohol consumption, uncontrolled hypertension and one interaction at a time.

CI indicates confidence interval; NSAID, nonsteroidal anti-inflammatory drug.

Table S13. Major bleeding in the XANTUS population stratified by HAS-BLED and ORBIT bleeding risk scores and validation of the XANTUS bleeding score using Harrell's C-index.

Score category	Overall n (%)	Patients with major bleeding* n (%)	Incidence proportion, % (95% CI)	Incidence rate Events per 100 years (95% CI)	Harrell's C of scores (individual score points) (95% CI)	Harrell's C of scores (categorizations) (95% CI)
**HAS-BLED score**†					0.59 (0.55–0.64)	0.56 (0.52–0.61)
Low (0)	318 (4.7)	1 (0.3)	0.31 (0.01–1.74)	0.36 (0.01–2.03)		
Medium (1–2)	4593 (67.7)	78 (1.7)	1.70 (1.34–2.11)	1.89 (1.49–2.35)		
High (≥3)	1854 (27.3)	48 (2.6)	2.59 (1.91–3.42)	2.88 (2.12–3.82)		
**ORBIT score**‡					0.62 (0.54–0.69)	0.59 (0.52–0.66)
Low (0–2)	1192 (17.6)	27 (2.3)	2.27 (1.50–3.28)	2.61 (1.72–3.80)		
Medium (3)	270 (4.0)	13 (4.8)	4.81 (2.59–8.09)	5.89 (3.13–10.07)		
High (4–7)	240 (3.5)	11 (4.6)	4.58 (2.31–8.05)	5.54 (2.76–9.91)		

Number of patients and major bleeding events from the XANTUS study were stratified by HAS-BLED and ORBIT bleeding risk scores. The score for a patient is unknown if the information on one of the components of the score is missing.

*Treatment emergent adjudicated.

†Risk factors: **H**ypertension (uncontrolled), **A**bnormal renal and liver function (one point each), **S**troke, **B**leeding (history or predisposition (anemia)), **L**abile INRs, **E**lderly (>65 years), Drugs or alcohol (one point for antiplatelet agents, other anticoagulants or NSAIDs; one point for alcohol excess).

‡Risk factors: Older age (75 years or older), Reduced hemoglobin (<13 mg/dL in men and <12 mg/dL in women), hematocrit (<40% in men and <36% in women) or history of anemia, Bleeding history, Insufficient kidney function (eGFR <60 mg/dL/1.73 m^2), Treatment with antiplatelets.

CI indicates confidence interval; eGFR, estimated glomerular filtration rate; INR, international normalized ratio; NSAID, nonsteroidal anti-inflammatory drug.

Figure S1. **Graphical assessment of the proportional hazards assumption of the risk factors.**

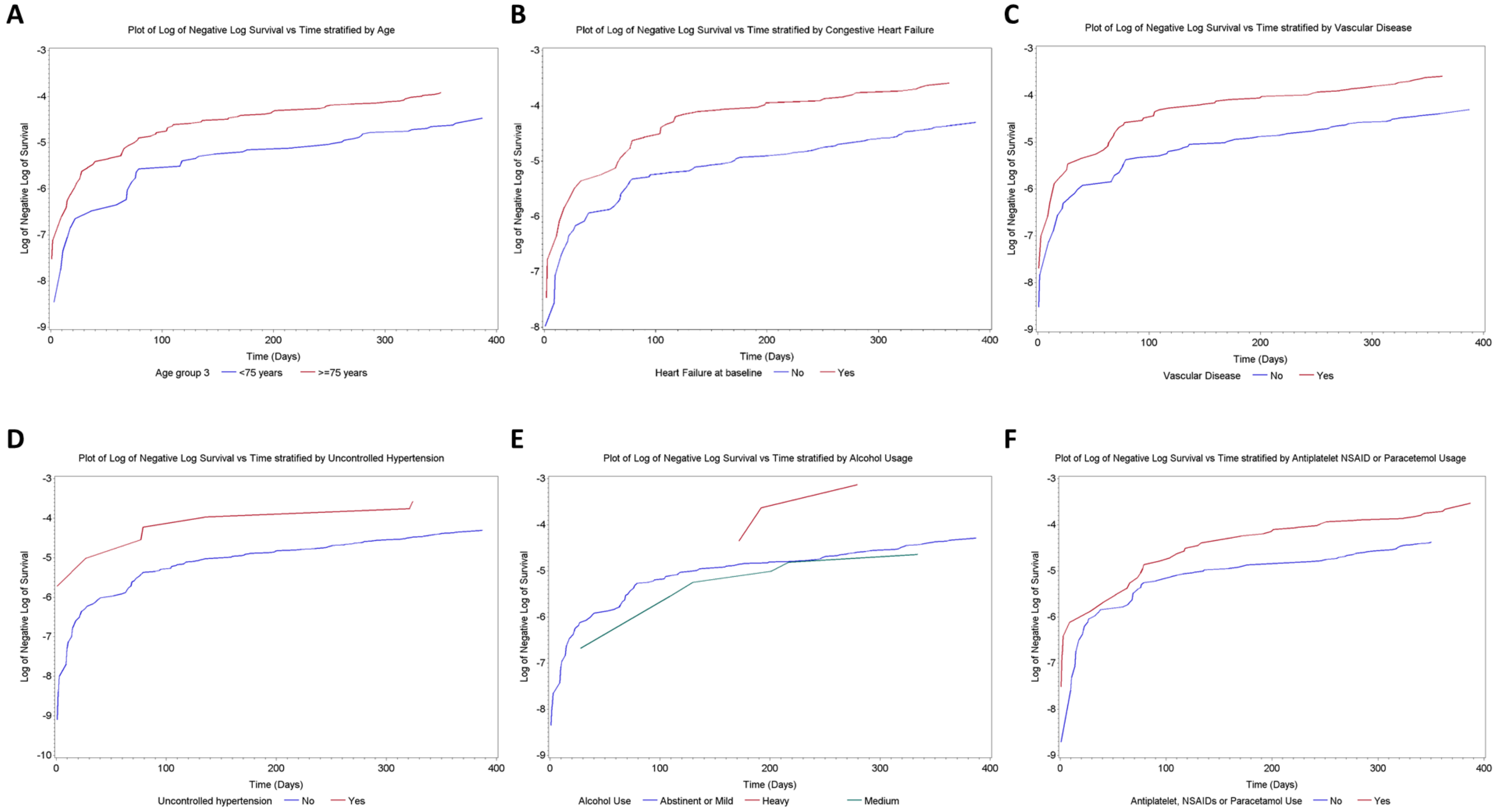

A, age; **B,** congestive heart failure; **C,** vascular disease; **D,** uncontrolled hypertension; **E,** alcohol usage; and **F,** NSAID or paracetamol usage.

NSAID indicates nonsteroidal anti-inflammatory drug.

Figure S2. Assessment of linearity for age – smoothed plot of Martingale residuals.

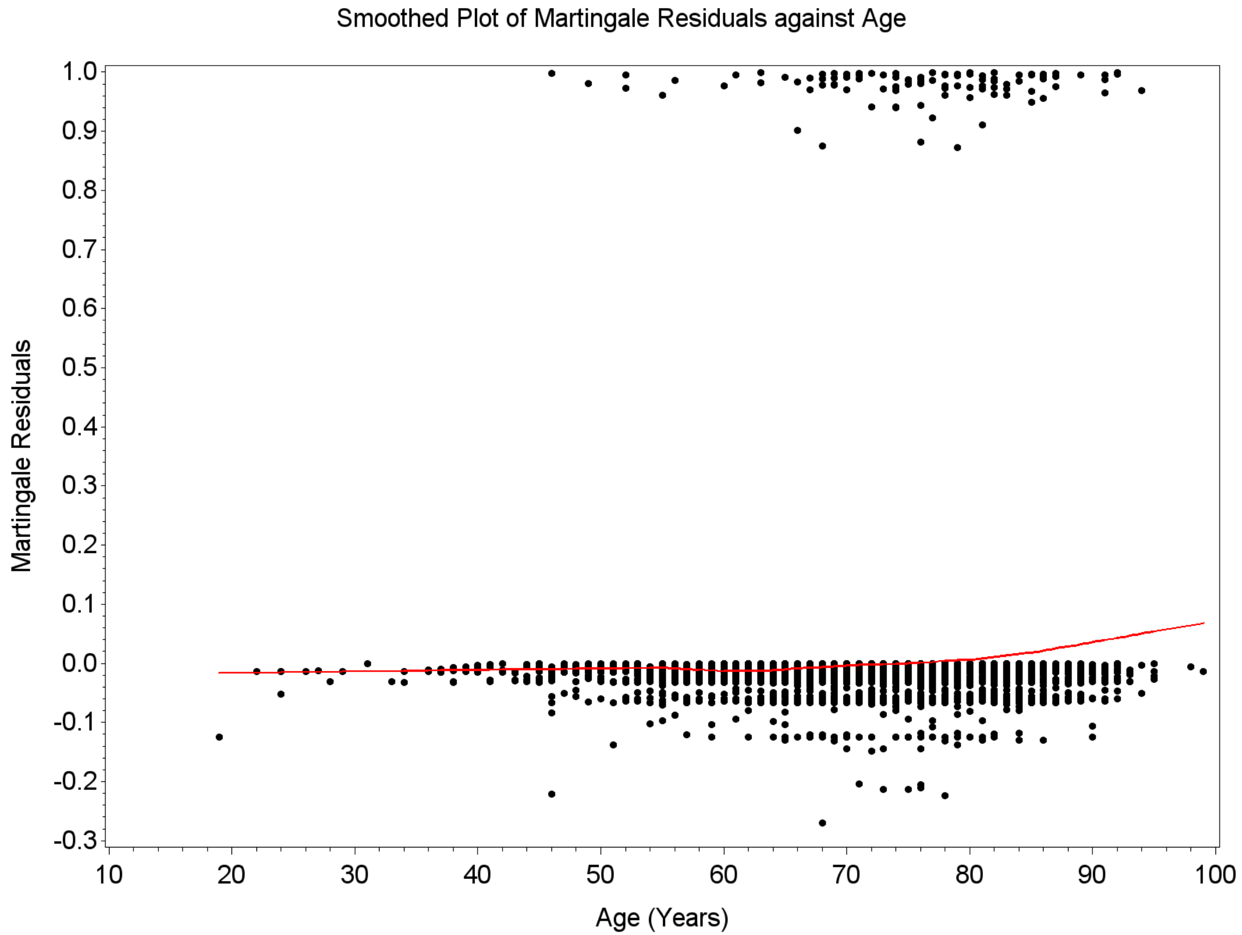

Figure S3. Calibration plot assessing correlation between actual and predicted probabilities in the final multivariate model.

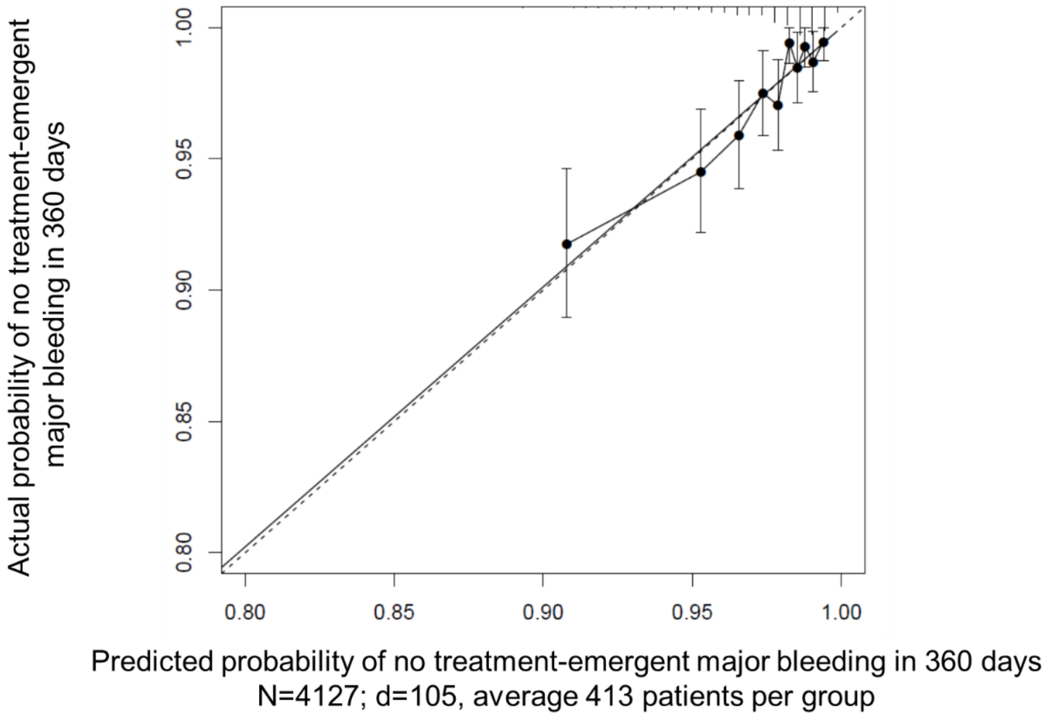

d indicates number of patients with an event; N, number of patients.
